# Long non-coding RNA HOTAIR: from pan-cancer analysis to colorectal cancer-related uridine metabolism

**DOI:** 10.18632/aging.205781

**Published:** 2024-05-01

**Authors:** Xuyu Chen, Siying Wang, Xin Jiang, Min Zhang, Yanbing Ding

**Affiliations:** 1 Department of Gastroenterology, The Affiliated Hospital of Yangzhou University, Yangzhou University, Yangzhou, China; 2 Department of Oncology, The Second Affiliated Hospital of Nanjing Medical University, Nanjing, China

**Keywords:** HOTAIR, pan-cancer, biomarker, colorectal cancer, uridine bypass

## Abstract

Long non-coding RNAs (lncRNAs) are involved significantly in the development of human cancers. lncRNA HOTAIR has been reported to play an oncogenic role in many human cancers. Its specific regulatory role is still elusive. And it might have enormous potential to interpret the malignant progression of tumors in a broader perspective, that is, in pan-cancer. We comprehensively investigated the effect of HOTAIR expression on tumor prognosis across human malignancies by analyzing multiple cancer-related databases like The Cancer Genome Atlas (TCGA) and Tumor Immune Estimation Resource (TIMER). Bioinformatics data indicated that HOTAIR was overexpressed in most of these human malignancies and was significantly associated with the prognosis of patients with cancer, especially in colorectal cancer (CRC). Subsequently, this study further clarified the utility of HOTAIR that downregulation of its expression could result in reduced proliferation and invasion of CRC cells. Mechanistically, HOTAIR upregulated the metabolic enzymes UPP1 by recruiting histone methyltransferase EZH2, thereby increasing the tumor progression. Our results highlight the essential role of HOTAIR in pan-cancer and uridine bypass, suggesting that the HOTAIR/EZH2/UPP1 axis might be a novel target for overcoming CRC. We anticipate that the role of HOTAIR in metabolism could be important in the context of CRC and even exploited for therapeutic purposes.

## INTRODUCTION

Cancer is a major public health problem with high global mortality and is the second leading cause of death worldwide. Despite tremendous breakthroughs in the field of tumors now, most cancer remains incurable [1]. Although the overall mortality rate has been effectively controlled due to the development of pathogenesis, diagnosis and treatment, the understanding of its biological characteristics still does not meet the requirements of translational medicine, with the result that the annual incidence rate continues to increase [2, 3]. Given the increased mortality in cancer patients, there is a need to further explore the mechanisms of cancer to improve prognosis and level of treatment. Through public data analysis and basic experimental exploration, we can now have a more comprehensive understanding of the important role of certain genes in human cancer progression.

Long non-coding RNA (lncRNAs) is an RNA molecule that is longer than 200 nucleotides and does not have the ability to encode proteins, playing a central role in different cancers through various potential molecular mechanism [4]. HOTAIR is located on chromosome 12 and is transcribed from the HOXC locus [5]. According to current research, HOTAIR regulates downstream gene expression by recruitment of chromatin modifiers and either by competitive binding with translation factor at the transcriptional and/or posttranscriptional levels [6, 7]. HOTAIR is an oncogenic RNA whose expression level is associated with many clinical features such as tumor grade and prognosis and has been explored to be oncogenic in colorectal cancer (CRC). HOTAIR also regulates various downstream protein expression via various mechanism axis. Enhancer of zeste homolog 2 (EZH2), a histone methyltransferase, regulate downstream target genes expression via specifically inducing the methylation on Lysine-27 of histone 3 (H3K27me) in multiple cancers [8, 9]. Uridine phosphorylase 1 (UPP1) functions in its phosphorolysis of uridine to uracil and ribose-1-phosphate, which is considered as an indispensable metabolic enzyme in the pyrimidine salvage biological processes and is upregulated in many cancers, including CRC [10]. When glucose is limiting, uridine might serve as alternative sources fulfil energy requirements. Uridine bypass makes CRC cells capable of using uridine and promoting tumor growth [11].

In conclusion, we conducted a comprehensive summary about the biological functions of HOTAIR with pan-cancers. The results show that HOTAIR is highly expressed in most tumors indicating that HOTAIR is linked to cancer development and prognosis. Furthermore, the expression level of HOTAIR also can reflect the immune infiltration level of tumor tissues. In CRC, HOTAIR upregulated the metabolic enzymes UPP1 by recruiting EZH2, thereby promoting the tumor progression, suggesting a novel metabolic axis for CRC therapy.

## MATERIALS AND METHODS

### Data collection

We used Gent2 database (http://gent2.appex.kr/gent2/), TIMER database (http://timer.cistrome.org/), Gene Expression Profiling Interactive Analysis (GEPIA) database (http://gepia2.cancer-pku.cn/#index) to compare the expression of HOTAIR between tumor tissues and normal tissues among 33 types of cancers. We obtained the overall survival data to investigate the association between the expression level of HOTAIR and survival status by Kaplan–Meier Plotter across various tumors in the GEPIA database and PrognoScan database. The cutoff value was set as cutoff-high (50%) and cutoff-low (50%) in distinguishing the high or low expression of HOTAIR. cBioPortal tool was used to analyze the condition of alteration frequency, mutated site information and mutation pattern of HOTAIR across all tumors. The infiltration data were used to demonstrate if there was a link between HOTAIR expression and infiltration in TIMER database. We used the TIMER and GEPIA database to explore the co-expression genes of HOTAIR, then performed gene ontology (GO) and Kyoto encyclopedia of genes and genomes (KEGG) pathway analysis in HOTAIR and neighborhood genes.

### Gent2 database

We used Gent2 database to explore the expression level of HOTAIR in pan-cancer level [12]. The database can reflect the genes expression in pan-cancer.

### TIMER database

TIMER2 is a database for analysis of immune infiltrates in various cancer types. TIMER2 provides immune infiltrates’ abundances estimated from the TCGA database to explore the immunological characteristics of tumors. The infiltration data were used to demonstrate if there was a link between the expression level of HOTAIR and infiltration [13].

### GEPIA database

GEPIA2 is a tool containing differential expression gene data and clinical data to assess the prognostic value of specific genes. Patient cases were divided into high expression and low expression depending on the gene expression level [14]. Hypothesis testing used the log-rank test.

### PrognoScan database survival

We got the overall survival data to investigate the clinical links between HOTAIR expression level and survival status by the PrognoScan database [15].

### The cBioPortal database

cBioPortal tool (14) was used to analyze the condition of alteration frequency, mutated site information and mutation pattern of HPOTAIR across TCGA tumors [16].

### LinkedOmics database

The LinkedOmics is a database that includes 32 TCGA cancer cohorts [17]. In our study, LinkedOmics database was used to explore genes differentially expressed with HOTAIR in the TCGA COAD cohort. The results were represented by volcano plots and heat maps.

### Gene enrichment analysis

We obtained the top 200 genes with a co-expression pattern to HOTAIR in all TCGA tumor tissue data in the “Similar Genes Detection” module of GEPIA2.

### Clinical tissue data

Twenty-five pairs of tumor and normal tissue samples were collected from patients with CRC. Tissue samples obtained by surgical treatment are frozen in liquid nitrogen. None of the patients had received specific treatment. CRC diagnosis was confirmed via pathological examination. All the informed consent forms of patients are signed and ethical approval for this experiment was approved by the Nanjing Medical University, Medical Ethics Committee.

### Cell culture, transfection and qRT- PCR

The COAD cell lines (SW480, SW620, HCT-116, DLD1, LOVO and HT-29) and the normal cell line (NCM460) were all obtained from Nanjing medical university laboratory. All cell lines were authenticated by STR profiling and were routinely tested for mycoplasma contamination. Cells were cultured in suitable culture media added with 1% penicillin/streptomycin and 10% fetal bovine serum (FBS). siRNAs (HIPPOBIO Biotechnology, Nanjing, China) of HOTAIR transfected in the SW480 and HCT-116 cell lines, followed the manufacturer’s instructions. Gene knockdown was achieved by transfecting cells with specific siRNAs or shRNA using Lipofectamine 3000 (Invitrogen, USA). The total RNA was extracted using TRIzol reagent (Invitrogen, USA). The expression levels of target genes and GAPDH were explored by qRT-PCR utilizing the SYBR qPCR (Vazyme, Nanjing, China). The primers were listed in Supplementary Data 1.

### Plasmid construction

Three UPP1 over-expressing plasmid and shRNAs targeting UPP1 were designed and purchased from GeneCopoeia Company (USA). They were transfected into CRC cells with lipo3000 (Invitrogen, USA) following the manufacturer’s manual. The sequences of all shRNAs were summarized in Supplementary Data 1.

### Cell proliferation assays

First, SW480 (1500 cells/well) and HCT-116 (1000 cells/well) cells were seeded in 96-well plates after cell transfection. CCK-8 reagent (Vazyme, Nanjing, China) was added into each well and incubated at 37°C for 2 h, the absorbance was measured at 450 nm using a microplate reader. CRC cells were planted into 6-well plates to perform colony formation assays. After a 2-week incubation period, we used 4% paraformaldehyde to fix the cells which were stained with crystal violet.

### Transwell assay

CRC cells were seeded in the plate upper chambers. The bottom chamber was added with full medium supplemented with 10% FBS. Following incubation at 24 hours, we used 4% paraformaldehyde fixing the cells on the lower surface and crystal violet staining the cells. Subsequently, cells were photographed under a microscope.

### ChIP assay and RIP assay

Chromatin immunoprecipitation (ChIP) assay and RNA immunoprecipitation (RIP) assay was conducted following manufacturer’s instruction (Millipore, USA). The sequences of primers for the UPP1 promoter were summarized in Supplementary Data 1.

### Western blot assay

We extracted total protein from CRC cells using RIPA buffer added with protease inhibitor. SDS–PAGE was used to separate proteins which was transferred onto PVDF membranes. Specific antibodies: UPP1 (Abcam, UK; ab128854), EZH2 (Proteintech, China; 21800-1-AP), Actin antibody was used as a control.

### Statistical analysis

All experiments were conducted using GraphPad Prism 8, and the results were expressed as means ± SD derived from at least three independent samples. To assess differences, an unpaired two-tailed *t*-test, as recommended for independent analysis, was employed. Statistical significance was determined by a *p*-value below 0.05.

## RESULTS

### Expression levels of HOTAIR in the pan-cancer analysis

**Figure 1:**
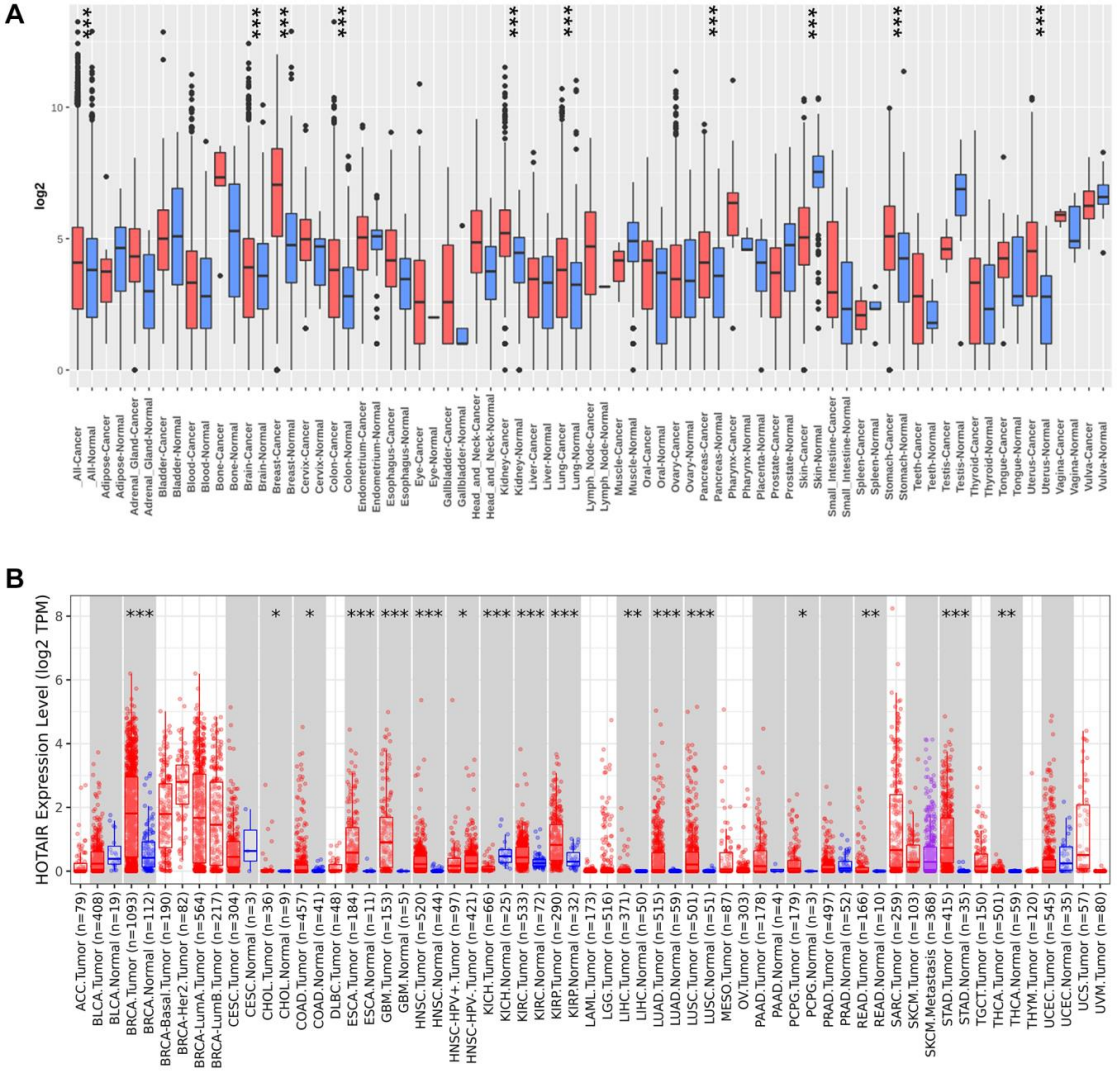
**The expression of HOTAIR in pan-cancer.** (**A**) Increased or decreased HOTAIR in datasets of different cancers compared with normal tissues in the Gent2 database; (**B**) HOTAIR expression in different cancers from TIMER2.0. ^*^p < 0.05; ^**^p < 0.01; ^***^p < 0.001.

We conducted a comparative analysis to assess HOTAIR expression in pan-cancer by comparing it between tumor and normal samples by the Gent2 database (Figure 1A). Compared with normal tissues, HOTAIR expression level was upregulated in various tumors, including colorectal cancer, breast cancer, brain cancer, kidney cancer, lung cancer, pancreatic cancer, skin cancer, stomach cancer and uterus cancer (*P* < 0.05). In addition, HOTAIR expression level was decreased in skin cancer (*P* < 0.05). Second, we analyzed the different expression level in HOTAIR using the TIMER database (Figure 1B). Figure 1B showed that overexpression of HOTAIR was observed in 17 types of tumors including colon adenocarcinoma (COAD), cholangiocarcinoma (CHOL), breast invasive carcinoma (BRCA), esophageal carcinoma (ESCA), glioblastoma multiforme (GBM), head and neck squamous cell carcinoma (HNSC), kidney renal clear cell carcinoma (KIRC), kidney renal papillary cell carcinoma (KIRP), liver hepatocellular carcinoma (LIHC), lung adenocarcinoma (LUAD), lung squamous cell carcinoma (LUSC), Pheochromocytoma and Paraganglioma (PCPG), rectum adenocarcinoma (READ), stomach adenocarcinoma (STAD) and thyroid carcinoma (THCA). However, the expression level of HOTAIR was reduced in Kidney Chromophobe (KICH) (all *P* < 0.05). Furthermore, we utilized the GEPIA dataset to investigate HOTAIR expression across human tumors (Supplementary Figure 1). Our findings revealed that HOTAIR expression level was higher in BRCA compared to neighboring non-cancerous tissues (*P* < 0.05; Supplementary Figure 2).

### Associations between the expression of HOTAIR and clinicopathological features progression

**Figure 2:**
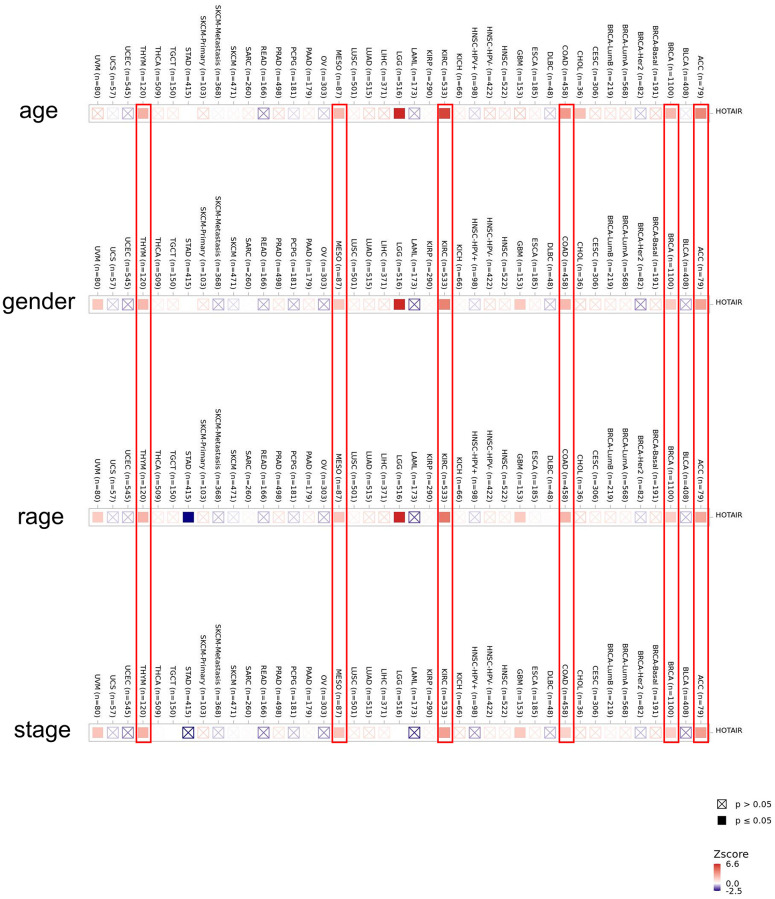
**Correlation between HOTAIR expression level and clinicopathological parameters in human cancers including age, gender, race and stage across all tumors in TCGA.** The red color indicates a positive correlation (0–1), while the blue color represents a negative correlation (−1–0). The correlation with *P*-value < 0.05 is considered as statistically significant. Statistically non-significant correlations values are marked with a cross.

We analyzed the expression level of HOTAIR at different pathology stages via the GEPIA database (Supplementary Figure 3). The HOTAIR expression varied significantly in COAD and READ (*P* < 0.05). We further explored the correlation between the RNA expression level and patients’ clinicopathological parameters in various cancers. We found a positive correlation between the age, gender, race and tumor stage and expression level of HOTAIR at the same time in ACC, BRCA, COAD, KIRC, MESO and THYM (Figure 2). These results showed that HOTAIR expression level could impact the prognosis in patients. To analyse the diagnostic value of HOTAIR, this study calculated and plotted the diagnostic receiver operating characteristic (ROC). We used the data from TCGA dataset plotting the diagnostic ROC for pan-cancer analysis (Supplementary Figure 4). Results showed that the area under the curve (AUC) of HOTAIR diagnostic ROC was 0.794 in BRCA, 0.829 in KICH, 0.661 in KIRC, 0.705 in LUAD, 0.847 in LUSC, 0.759 in PRAD, 0.934 in STAD, 0.648 in COAD, and 0.846 in PAAD indicating that HOTAIR may be a promising diagnostic biomarker in human cancers. Interestingly, we found in the results that the ROC curve was still well represented in the CRC. This result clearly reconfirmed the role of HOTAIR in clinical practice.

### Prognostic analysis of HOTAIR in human cancers

**Figure 3:**
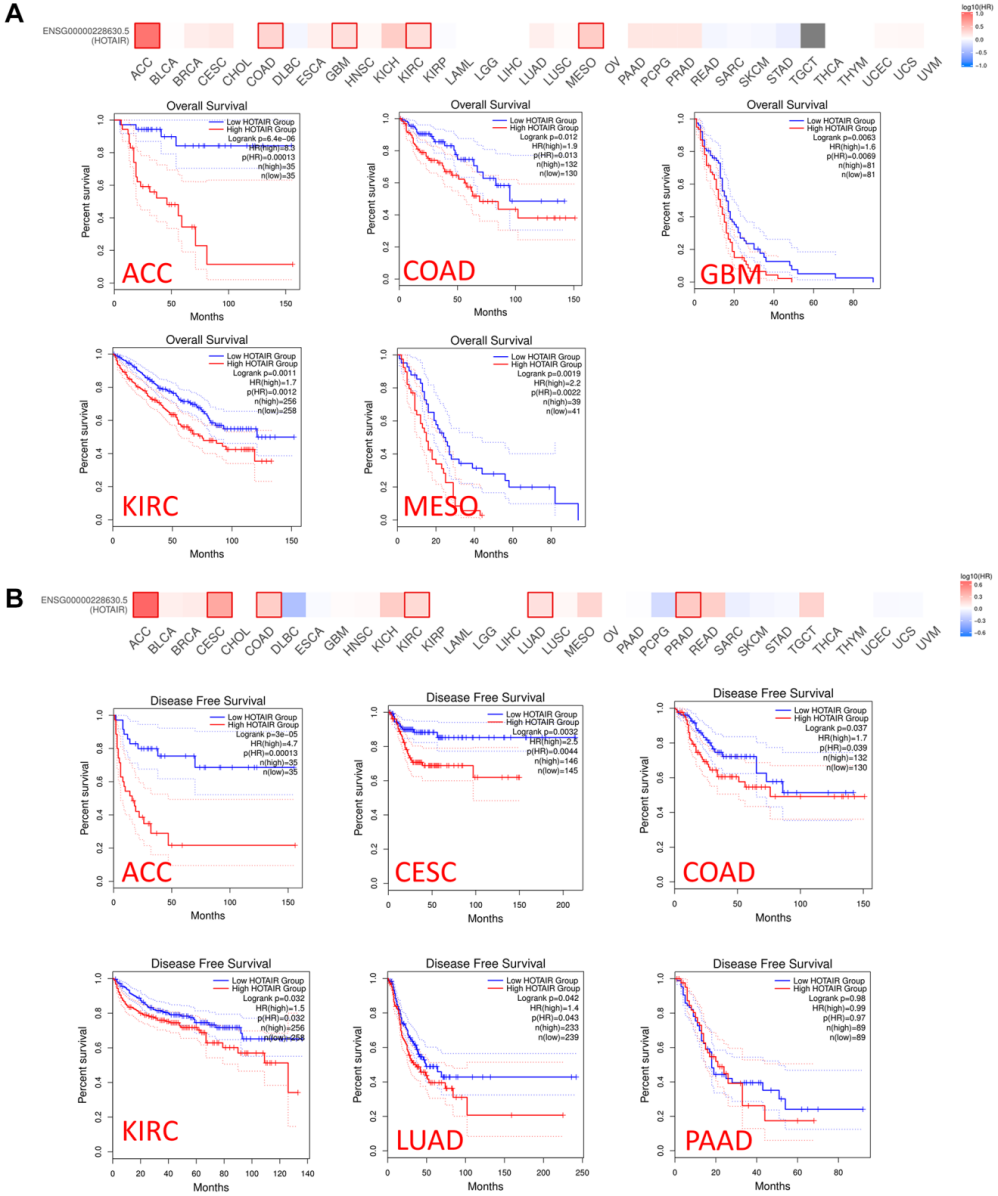
**Relationship between HOTAIR expression level and patient survival in TCGA tumors.** Relationship between HOTAIR gene expression and survival overall (**A**), disease-free survival (**B**) was assessed in all TCGA tumors using GEPIA2. The positive results of survival map and Kaplan-Meier curves are listed.

We conducted a prognosis analysis using data obtained from PrognoScan and GEPIA to examine the relationship between HOTAIR expression levels and overall survival (OS) across various cancer types. In PrognoScan database, the results revealed that HOTAIR expression associated with prognosis in colorectal and breast cancers. (Supplementary Figure 5A–5D). Furthermore, using the GEPIA database, we found that high HOTAIR expression in adrenocortical carcinoma (ACC), COAD and KIRC was associated with shorter OS and DFS (Figure 3A, 3B), indicating a detrimental effect of HOTAIR overexpression in these cancer types. Conversely, in GBM, mesothelioma (MESO), cervical squamous cell carcinoma and endocervical adenocarcinoma (CESC), LUAD and Pancreatic adenocarcinoma (PAAD), overexpression of HOTAIR was associated with longer OS, suggesting a potentially protective role of HOTAIR in these specific cancer contexts. These findings highlight the complexity and background dependence of the role of HOTAIR in tumorigenesis and progression, whose function exhibits completely opposite properties in different tumor types. The internal mechanisms controlling these different effects are likely to be extremely complex and require further investigation.

### Genetic alterations of HOTAIR

We used the cBioPortal online database to conduct an analysis of genetic alterations in HOTAIR across TCGA types. We explored that HOTAIR was altered in 2.7% patients including amplification and deep deletion (Supplementary Figure 6A). Among the alterations, the most common type was amplification. The most prevalent genetic alteration in HOTAIR was the “amplification” type, observed in nearly all cancer types. (Supplementary Figure 6B). Notably, the highest frequency of HOTAIR alterations (10%) was observed in PAAD patients, primarily in the form of “amplification”. In addition, “deep deletion” of HOTAIR occurred in 1% of patients with BRCA, and mutations in HOTAIR were the deep deletion type in 0.5% of patients with LIHC. Next, we concluded the relationship between the types of the alterations and the levels of HOTAIR mRNA. As shown in Supplementary Figure 6C, among the alterations the level of HOTAIR mRNA increased from the deletion group, amplification group, gain group to the diploid group in a sequential manner.

### HOTAIR associated with the CNV events across human cancers

**Figure 4:**
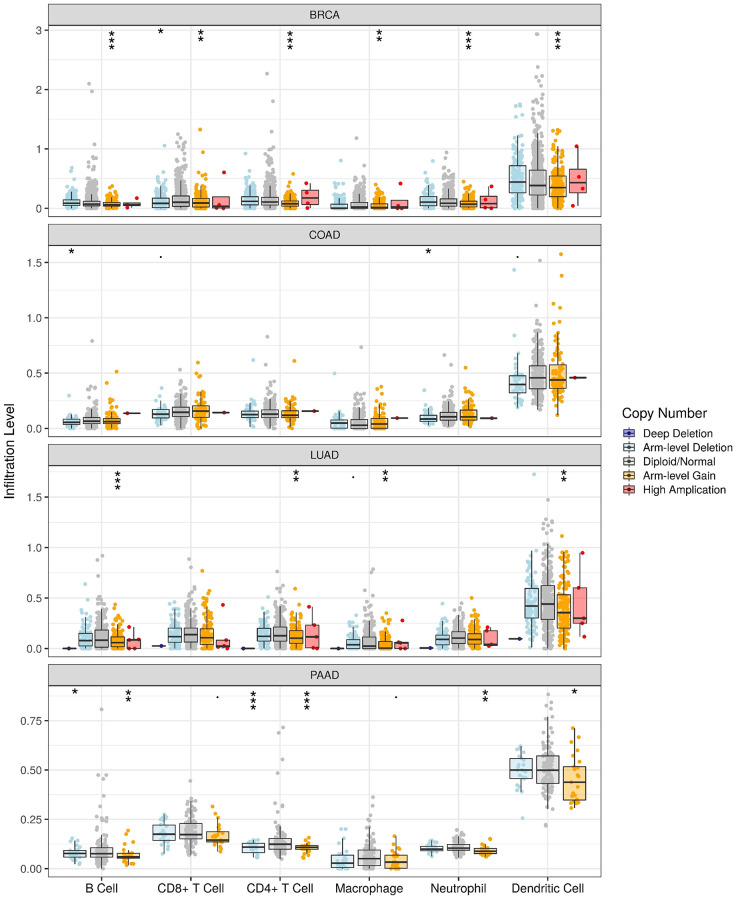
**TIMER revealed that expression of HOTAIR was associated with immune infiltrates.** HOTAIR CNV affects the infiltrating levels of various immune cells in BRCA, COAD, LUAD and PAAD. ^*^p < 0.05; ^**^p < 0.01; ^***^p < 0.001.

The progression of tumors is inseparable from the immune system, and the rise of immunotherapy is changing the paradigm of cancer treatment. Therefore, to examining the possibility of that HOTAIR might be explored as an immune target in cancer therapy, we took advantage of the TIMER database to investigate the association between the expression level of HOTAIR and immune infiltration by six main immune cells including B cells, CD8+ T cells, CD4+ T cells, macrophages, neutrophils, and dendritic cells (Figure 4). Among different cancers, HOTAIR CNV is linked to the infiltrating level of different types of immune cells: B cells, CD8+ T cells, CD4+ T cells, macrophages, neutrophils and dendritic cells in the BRCA, B cells and neutrophils in COAD, B cells, CD4+ T cells, macrophages and dendritic cells in the LUAD and B cells, CD4+ T cells, neutrophils and dendritic cells in the PAAD. Thus, HOTAIR may affect the immunity of different cancers and do so through potential distinct mechanisms.

### Immune infiltration analysis

**Figure 5:**
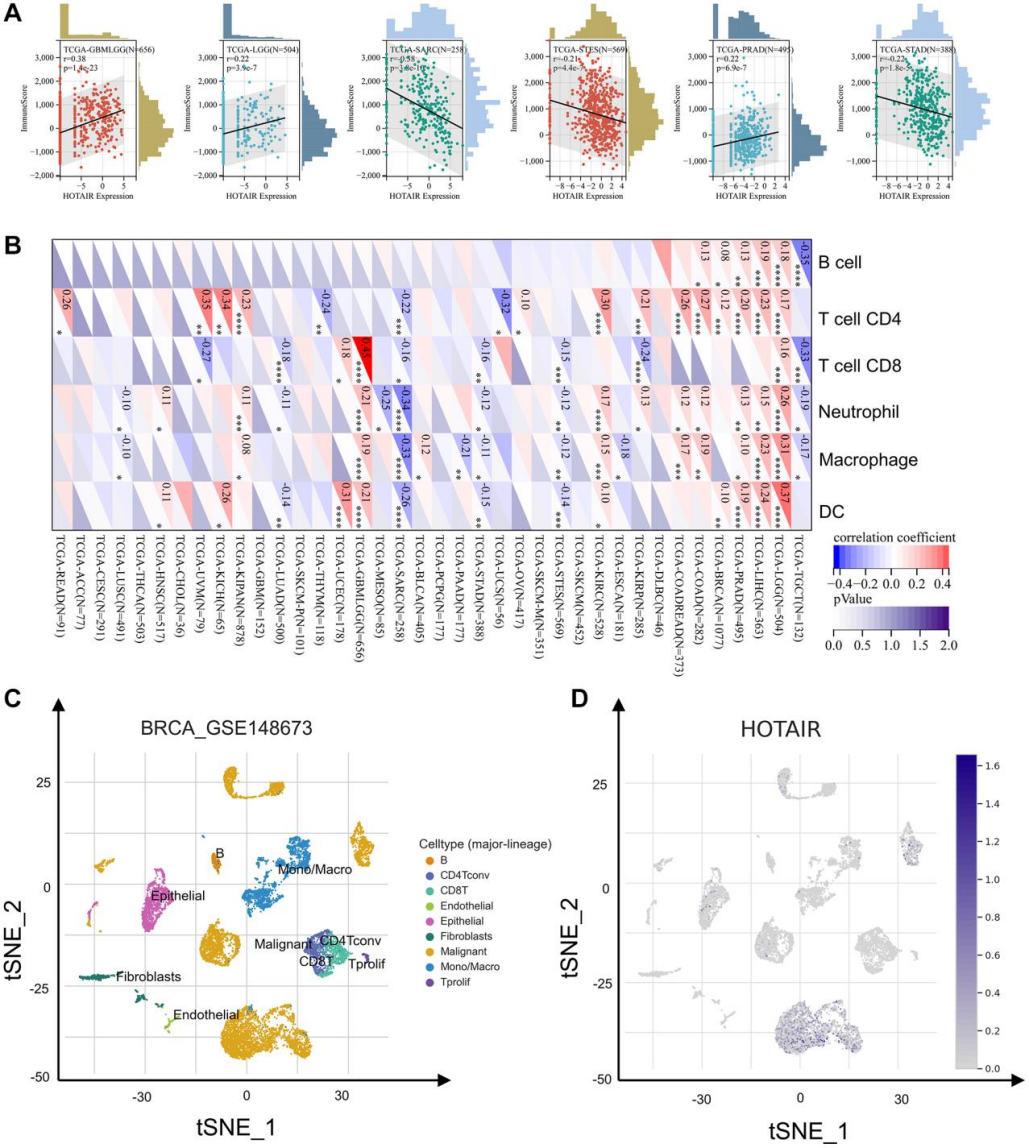
**The correlation of HOTAIR expression with immune infiltration levels.** (**A**) The correlation between HOTAIR and immune score. (**B**) The correlation between HOTAIR and immune cells. (**C**) Cells from the GSE148673 dataset were mapped on the tSNE plot. (**D**) tSNE plot illustrating HOTAIR expression profile at the cell level.

We explored the correlations between HOTAIR and immune infiltration. As shown in Figure 5A, in GBMLGG, LGG and PRAD, there is a positively correlation between the immune score and HOTAIR expression. Conversely, there is a negative correlation between the immune score and HOTAIR expression in SARC, STES and STAD. These results indicate a tight correlation between HOTAIR expression levels and tumor immune responsiveness in most tumor types (Figure 5B). In COAD, HOTAIR positively correlated with four immune cell types (B cells, neutrophils, CD4^+^ T cells and macrophages). From the single-cell level, HOTAIR expression level was most highly in malignant cells (Figure 5C). Figure 5D shows the close connection between high HOTAIR expression with relative percentage of cells.

### Gene enrichment analysis of HOTAIR

To elucidate the functional mechanism of HOTAIR in carcinogenesis, we employed the GEPIA tool to identify genes that exhibited co-expression with HOTAIR. A heatmap is presented to illustrate the positive correlation between HOTAIR and these 5 genes (HOXC10, HOXC11, HOXC12, HOXC13, and NKD2) (Supplementary Figure 7A). HOTAIR expression correlated positively with HOXC10 (homeobox C10) (R = 0.38), HOXC11 (homeobox C11) (R = 0.6), HOXC12 (homeobox C12) (R = 0.66), HOXC13 (homeobox C13) (R = 0.46) and NKD2 (NKD inhibitor of WNT signaling pathway 2) (R = 0.6) (all *p* < 0.001) (Supplementary Figure 7B). Furthermore, gene pathway enrichment analysis revealed that the biological processes involving the top HOTAIR-associated genes were closely associated with protein modifications-related pathways (Supplementary Figure 7C). The co-expression genes of HOTAIR were analyzed by LinkedOmics database to further explore the biological effects of HOTAIR. As shown in Supplementary Figure 7D, the expression level of HOTAIR affects the expression of a large number of genes. Genes positively (Supplementary Figure 7E) and negatively (Supplementary Figure 7F) both correlated with HOTAIR are shown in the heat map.

### LINC00467 promotes CRC cell proliferation and migration

**Figure 6:**
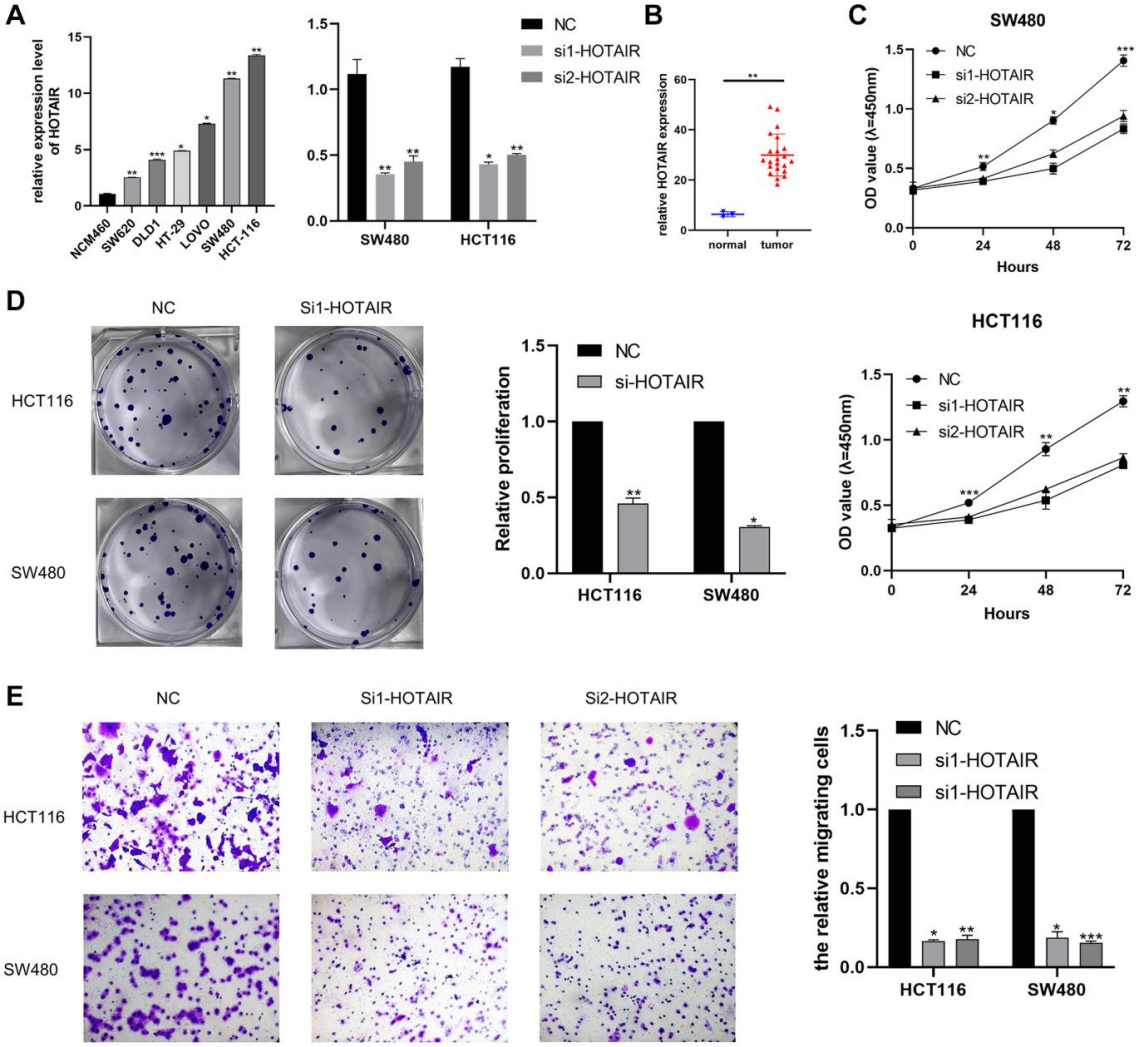
**Effect of HOTAIR in CRC cell proliferation and migration.** (**A**) Transcription level of HOTAIR in CRC cell lines and NCM460. (**B**) Transcription level of HOTAIR in HCT116 and SW480 were significantly downregulated by si-HOTAIR transfection, respectively. (**C**) Relative expression of HOTAIR in CRC tissues was assessed by comparing with normal samples (*n* = 25). (**D**) Cell proliferation was assessed by CCK assay. (**E**) Transwell assay employed to detect the migration ability of HOTAIR knockdown cells. ^*^p < 0.05; ^**^p < 0.01; ^***^p < 0.001.

Differential analysis and prognostic analysis showed that HOTAIR played an important biological role in CRC. By analyzing the TCGA dataset, we summarized the correlation between HOTAIR expression levels and clinical indicators such as age and stage (*p* < 0.05) (Table 1). First, we examined the expression level of HOTAIR in related CRC cell lines by RT-qPCR and HOTAIR expression was highly expressed in CRC cells compared with the normal cell line NCM460 (Figure 6A). Thus, we selected SW480 and HCT116 cell lines for further research. We also confirmed the knockdown efficacy of siRNAs (si1 and si2) targeting HOTAIR through RT-qPCR. Compared with adjacent normal tissues, LINC00467 expression level was upregulated in tumor tissue samples (Figure 6B). The proliferation assay (CCK-8 and colony assays) proved that the low expression of HOTAIR significantly suppressed cell proliferation (Figure 6C, 6D). Our study also demonstrated that HOTAIR siRNA suppressed the cell migration abilities in both SW480 and HCT116 cell lines (Figure 6E).

**Table 1:** Correlations between HOTAIR expression and clinicopathological characteristics in TCGA-COAD.

**Characteristic**	**Low expression of HOTAIR**	**High expression of HOTAIR**	***p***
* n*	322	322	
T stage, *n* (%)			0.180
T1	11 (1.7%)	9 (1.4%)	
T2	65 (10.1%)	46 (7.2%)	
T3	210 (32.8%)	226 (35.3%)	
T4	33 (5.1%)	41 (6.4%)	
N stage, *n* (%)			**0.004**
N0	201 (31.4%)	167 (26.1%)	
N1	75 (11.7%)	78 (12.2%)	
N2	44 (6.9%)	75 (11.7%)	
M stage, *n* (%)			0.103
M0	240 (42.6%)	235 (41.7%)	
M1	36 (6.4%)	53 (9.4%)	
Age, median (IQR)	66 (57, 74)	69 (59, 77)	**0.034**

### HOTAIR affects CRC progression through uridine bypass via EZH2/UPP1 axis

**Figure 7:**
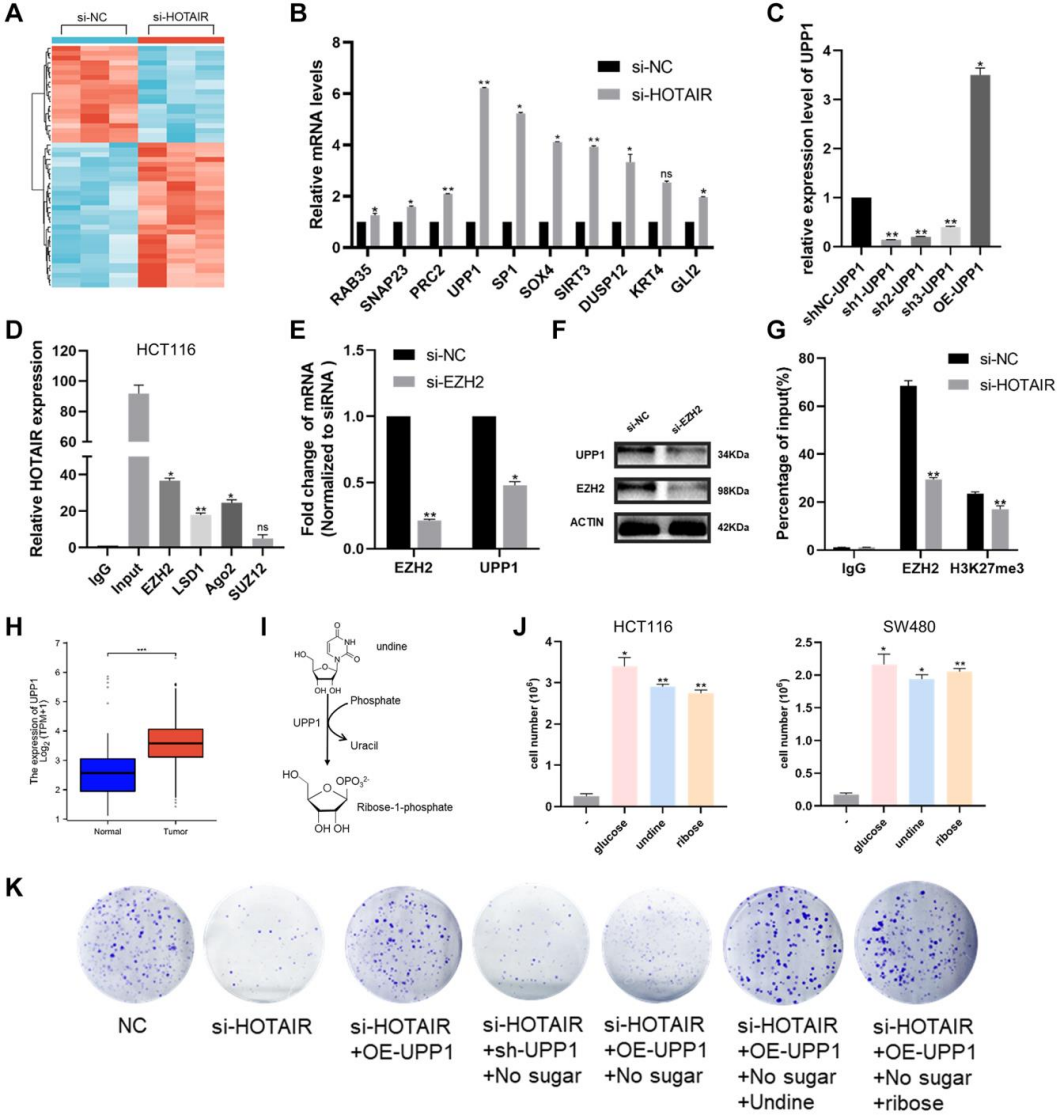
**HOTAIR affects CRC proliferation through uridine bypass via EZH2/UPP1 axis.** (**A**) A hierarchically clustered heatmap of differentially expressed genes in CRC cells after transfection of si-HOTAIR or NC-siRNAs. (**B**) Ten representative genes expression levels in CRC cells depleted of HOTAIR. (**C**) The knockdown and over-expressing efficiency of UPP1 were detected by RT-qPCR. (**D**) RIP assays were performed in CRC cells to show HOTAIR co-immunoprecipitation with EZH2, LSD1, Ago2 and SUZ12. (**E**, **F**) EZH2 and UPP1 mRNA and proteins level in CRC cells transfected with si-EZH2 or siRNA-NC by qRT-PCR and western blot analysis. (**G**) EZH2 occupancy on the UPP1 promoters was upregulated by HOTAIR knockdown by ChIP-qPCR assay. (**H**) UPP1 was highly expressed in CRC tissues compared with normal tissues through TCGA. (**I**) Reaction catalysed by UPP1 proteins. (**J**) Cell growth assays of HCT116 control cells in glucose-free media in the presence of 10 mM of either glucose, uridine or ribose. (**K**) Colony-forming assays assessed CRC cell proliferation in the presence of si-HOTAIR, sh-UPP1, OE-UPP1, glucose, uridine or ribose.

To further explore the mechanism of how HOTAIR regulates colorectal cancer proliferation and migration, we performed an RNA sequencing analysis (Figure 7A). Among the large number of differentially expressed genes, we selected the top 10 genes with the highest expression for experimental verification (Figure 7B). In the qRT-PCR analysis, we found the highest expression of UPP1 gene in the case of HOTAIR knockdown. Then we designed the HOTAIR expression vector and three shRNAs to study its biological role (Figure 7C). Studies have shown that lncRNAs can regulate the downstream genes expression, such as EZH2, Ago2, LSD1, and SUZ12, by interacting with RNA-binding proteins [18, 19]. So, we carried out RIP experiment to verify and screen and the results showed the strongest binding between HOTAIR and EZH2, indicating that HOTAIR interacted with EZH2 specifically (Figure 7D). Furthermore, we observed that UPP1 was down-regulated at both mRNA and protein levels when EZH2 was knocked down, indicating that EZH2 promoted the high expression of UPP1 (Figure 7E, 7F). Chromatin immunoprecipitation (Chip) assay showed that EZH2 directly bound to the UPP1 promoter region and induced H3K27 trimethylation. Meanwhile, knockdown of HOTAIR reduced EZH2 and H3K27 trimethylation levels through the UPP1 promoter (Figure 7G). These results showed that HOTAIR promotes CRC progression through the upregulate of UPP1 via interacting with EZH2. The expression level of UPP1 is significantly increased in CRC samples (Figure 7H). UPP1 is a uridine phosphorylase catalysing the phosphate-dependent catabolism of uridine into R1P and uracil (Figure 7I). Glucose oxidation drives cellular bioenergetics. Recent studies have confirmed that UPP1 liberates uridine-derived ribose to fuel tumor metabolism and thereby support cells proliferation [20]. This phenomenon depends on uridine being present and expression of UPP1 (Figure 7J). Colony-forming assays have also demonstrated that HOTAIR could affect CRC progression through uridine bypass via EZH2/UPP1 axis (Figure 7K).

## DISCUSSION

HOTAIR was discovered by Howard Chang’s group that repressors the expression of homeobox gene D cluster (HOXD) by recruiting a transcriptional corepressor [21]. The human HOTAIR gene is located in the intergenic region between HOXC11 and HOXC12 in the HOXC gene cluster on chromosome 12 [22]. Since the first appearance of HOTAIR in the field of oncology, its highly expressed role has been considered as an oncogene [23–26]. For example, HOTAIR contributes to 5FU Resistance through suppressing miR-218 and activating NF-κB/TS signaling in colorectal cancer [27]. Consistent with the results of our study, HOTAIR was highly expressed in cancer tissues and positively correlated with cancer proliferation and migration in multiple tumor cohorts. Further studies with a larger sample size will help to draw reliable conclusions about the expression of HOTAIR in tumor tissues. Although more and more studies have shown that HOTAIR has important clinical implications in a variety of tumors [28–30], Up to now, the role of HOTAIR in pan-cancer has not been reported. Therefore, we need to better understand the role of HOTAIR in pan-cancer and its potential prognostic value, as well as the molecular mechanism of its action.

We found that HOTAIR genes are highly expressed in a total of seventeen cancers and lowly expressed in one cancer by TIMER database. The differences in samples led to the differences in the results of different databases. Sample differences lead to differences in the results of different databases, but as long as there are enough samples, the cancer-promoting role of HOTAIR is still plausible. Despite great progress has been achieved in recent years, the mortality of CRC is still increasing [31]. Effective prognostic prediction is of great significance to improve the survival of patients with CRC. However, to date, prognostic biomarkers remain limited. High expression of HOTAIR in CRC was identified, and a high expression of HOTAIR was associated with poor survival in patients with CRC. Interestingly, the pan-cancer analysis also revealed a markedly poor survival in tumors with high HOTAIR expression, which indicated that HOTAIR might participate in tumorigenesis. Through genetic alteration analysis of HOTAIR, we observed that HOTAIR produced significant alterations in a variety of cancers. Therefore, we can hypothesize that HOTAIR expression and mutation are potential parts of tumor biology.

The relationship between immunity cells and cancer cells is very close and immunotherapy represents the future of clinical cancer treatment [32].

Given the important role of HOTAIR in a variety of tumors, we further explored the correlation between tumor immunity and HOTAIR. Accumulating evidence suggests that HOTAIR is closely related to the proliferation and metastasis of human cancers [33, 34]. However, according to the current research, there are few studies on the relationship between HOTAIR expression level and immune infiltration.

We observed a significant correlation between HOTAIR expression and tumor immune cell infiltration, including B cells, CD8+ T cells, CD4+ T cells, macrophages, neutrophils, and dendritic cells. This suggests that HOTAIR may impact tumor processing and prognosis through its influence on cancer immunity. In this study, we provide the first statistical evidence of a positive association between HOTAIR expression and the infiltration level of CD4+ T cells in COAD. Pre-clinical and clinical investigations have identified intra-tumoral cytotoxic CD4+ T cells capable of directly eliminating cancerous cells [35]. Studies have demonstrated that tumor-derived exosomes facilitate the immunosuppressive function of B-regs by delivering HOTAIR in COAD. Tumor-derived HOTAIR directs B cells towards a regulatory phenotype characterized by programmed cell death-ligand 1 (PDL1) expression in CRC, thereby inducing PDL1-expressing B cells to suppress CD8+T cell activity [36]. The pivotal role of HOTAIR in both immunity and tumorigenesis necessitates further investigations to elucidate its biological functions and underlying mechanisms.

In our study, further investigation confirmed a robust positive correlation between HOTAIR expression and the HOXC cluster genes. The HOTAIR gene is transcribed in an antisense orientation relative to its flanking HOXC11 and HOXC12 genes. Previous studies have demonstrated that increased DNA methylation in an intergenic CpG island located between HOXC12 and HOTAIR is positively associated with HOTAIR expression [37]. Additionally, we observed that HOTAIR and its interacting genes primarily focus on “distal pattern formation”, “histone modification”, “covalent chromatin modification”, etc.

We then validated the function of HOTAIR in COAD and observed significant overexpression of HOTAIR, which was consistent with current research, using bioinformatics analysis. Furthermore, we explored the oncogenic role of HOTAIR through cell experiments and found that it potentiates cancer metastasis and tumor progression by recruiting EZH2 [18]. Similar to previous studies, we identified HOTAIR as an independent prognostic factor in CRC. Specifically, blocking HOTAIR with siRNA significantly suppressed CRC cancer cell growth and migration *in vitro*. In future studies, we aim to confirm the function of HOTAIR both *in vivo* and *in vitro* using more advanced molecular biology techniques. Glucose serves as a source of energy. When glucose is limiting, alternative nutrients such as undine could fuel ATP production and gluconeogenesis [38]. HOTAIR could affects CRC progression through uridine bypass via EZH2/UPP1 axis which upregulated UPP1 transcription level and uridine catabolism in CRC cells. We propose that a new signaling axis controlling UPP1 transcription thus maintaining the homeostasis of energy and biosynthesis.

While our study has improved our understanding of HOTAIR from a pan-cancer perspective, there are still several limitations. Firstly, the sample size of cancer patients in the TCGA database was significantly larger than that of normal patients. Secondly, the absence of clinical factors in the public database, such as specific details regarding medication and/or surgical treatment, also impacts patient prognosis. Additionally, our study does have a limitation concerning the association between HOTAIR and immunotherapy. The involvement of HOTAIR in immunotherapy should be further validated through clinical and cellular experiments, such as co-culturing tumor cells with immune cells using interference for HOTAIR. Lastly, this present study is retrospective, and prospective studies should be conducted to address the limitations inherent to retrospective research design. Despite these limitations, our study provides valuable insights into investigating the function of HOTAIR in cancers and identifies potential targets and prognostic markers for CRC treatment. In conclusion, our pan-cancer analysis offers a comprehensive overview of the oncogenic roles played by HOTAIR across various human cancers. Overexpression of HOTAIR generally indicates poor prognosis for cancer patients. Our study characterizes HOTAIR expression patterns across different cancer types and highlights its potential value as a predictive biomarker while shedding light on further exploration into its prognostic and therapeutic potential. Growing evidence that HOTAIR plays crucial roles in carcinogenesis. In the study, HOTAIR was demonstrated to promote tumorigenesis via recruiting EZH2 in CRC cells, indicating the clinical importance of HOTAIR/EZH2/UPP1 axis as a promising therapeutic target for CRC. Recently, the rapid progress in mRNA vaccine design and delivery technologies has significantly expedited the development and clinical application of mRNA-based cancer vaccines. Future investigations could potentially explore synergistic combinations of HOTAIR cancer vaccines with immunotherapeutic approaches to enhance the efficacy of cancer treatment.

## Supplementary Materials

Supplementary Figures

Supplementary Data 1
