# Supplementary material for: Long non-coding RNA HOTAIR: from pan-cancer analysis to colorectal cancer-related uridine metabolism
Source: Aging (Albany NY). 2024 May 1;16(9):7752–73. doi: 10.18632/aging.205781 (PMC11132002; doi:10.18632/aging.205781)
Supplement: Supplementary Figures [file aging-16-205781-s001.pdf]

SUPPLEMENTARY FIGURES

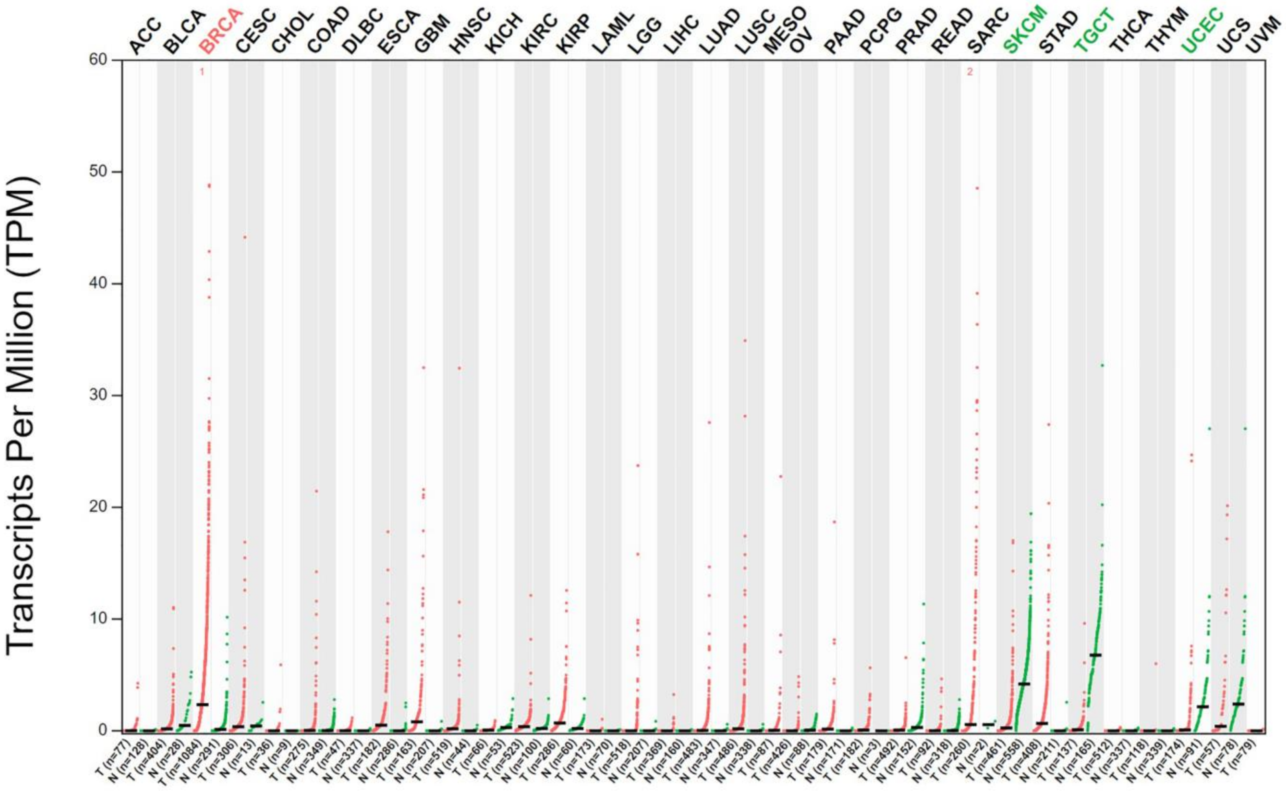

Supplementary Figure 1. The gene expression profile of HOTAIR across all tumor samples and paired normal tissues. Red and green spots represent cancer and normal tissues respectively. Red and green abbreviations of the cancers on the top represent a significant difference between cancer and noncancer. TCGA and GTEx data were plotted using GEPIA. A full list of the cancer type abbreviation can be found from GEPIA.

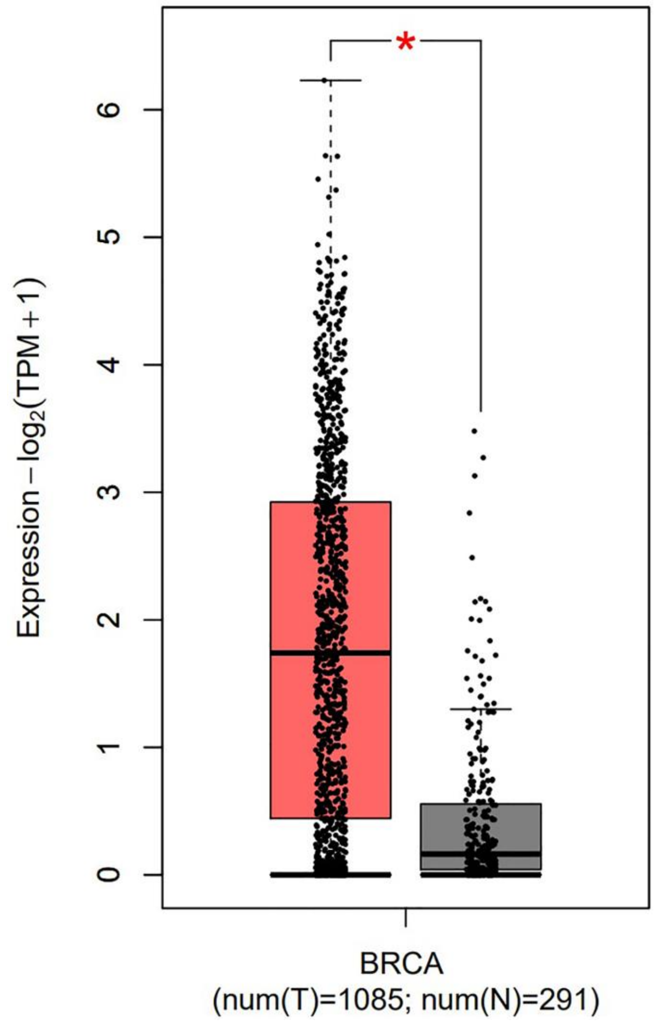

Supplementary Figure 2. Expression profile of the HOTAIR genes analyzed by GEPIA (normal = 291, tumor = 1085).

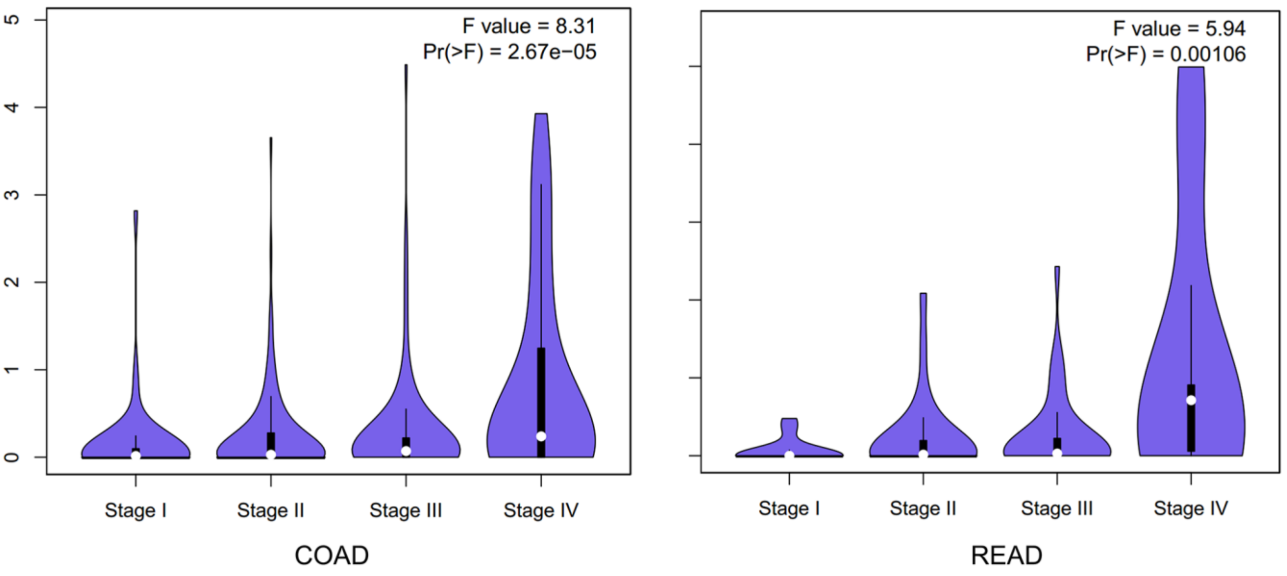

Supplementary Figure 3. Stage-dependent expression level of HOTAIR in CRC. Main pathological stages (stage I, stage II, stage III, and stage IV) of COAD and READ were assessed and compared using TCGA data. Expression levels are shown as Log2 (TPM+1).

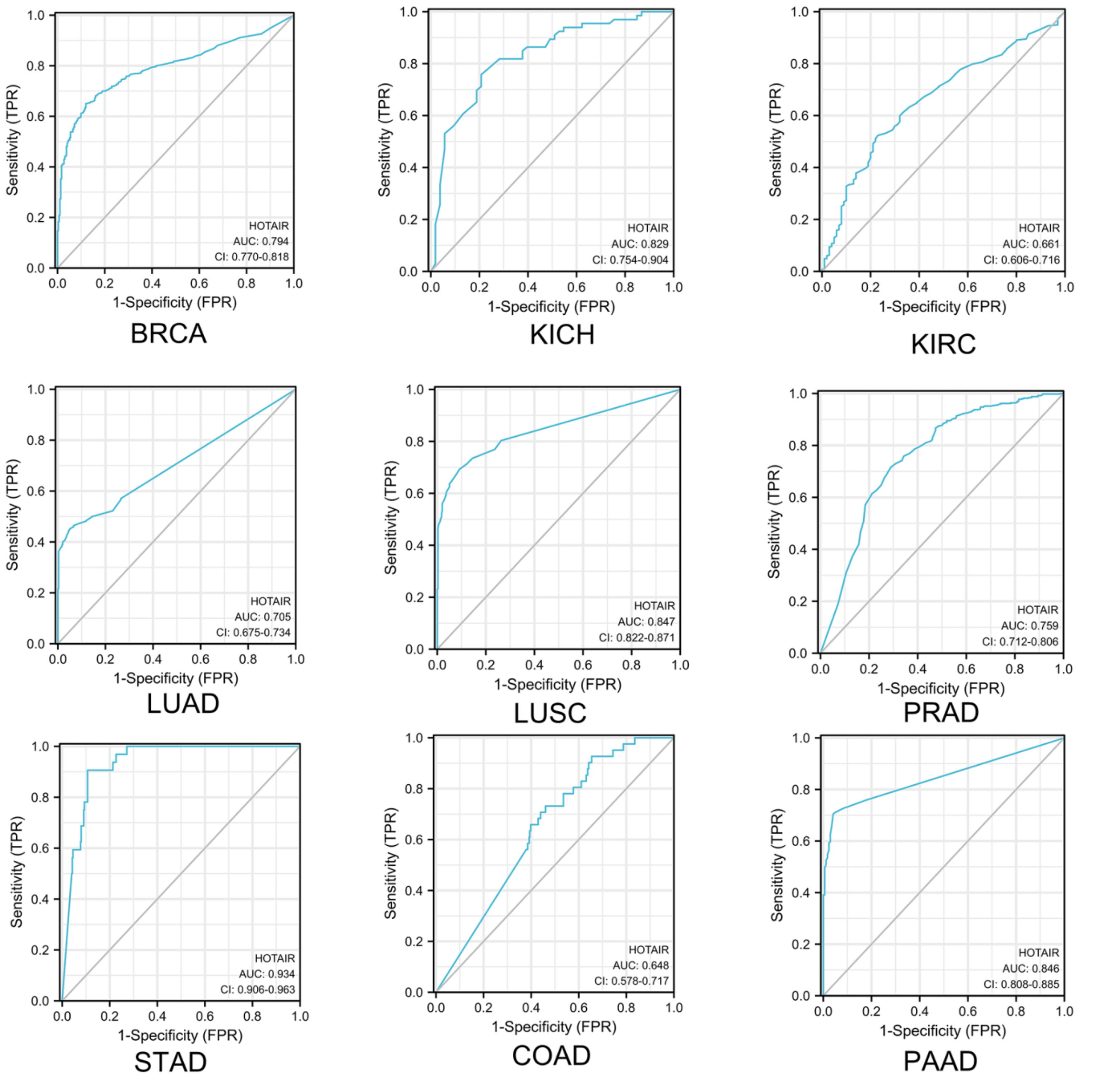

Supplementary Figure 4. Diagnostic value of HOTAIR in ROC curve. Diagnostic ROC of HOTAIR in BRCA, KICH, KIRC, LUAD, LUSC, PRAD, STAD, COAD and PAAD.

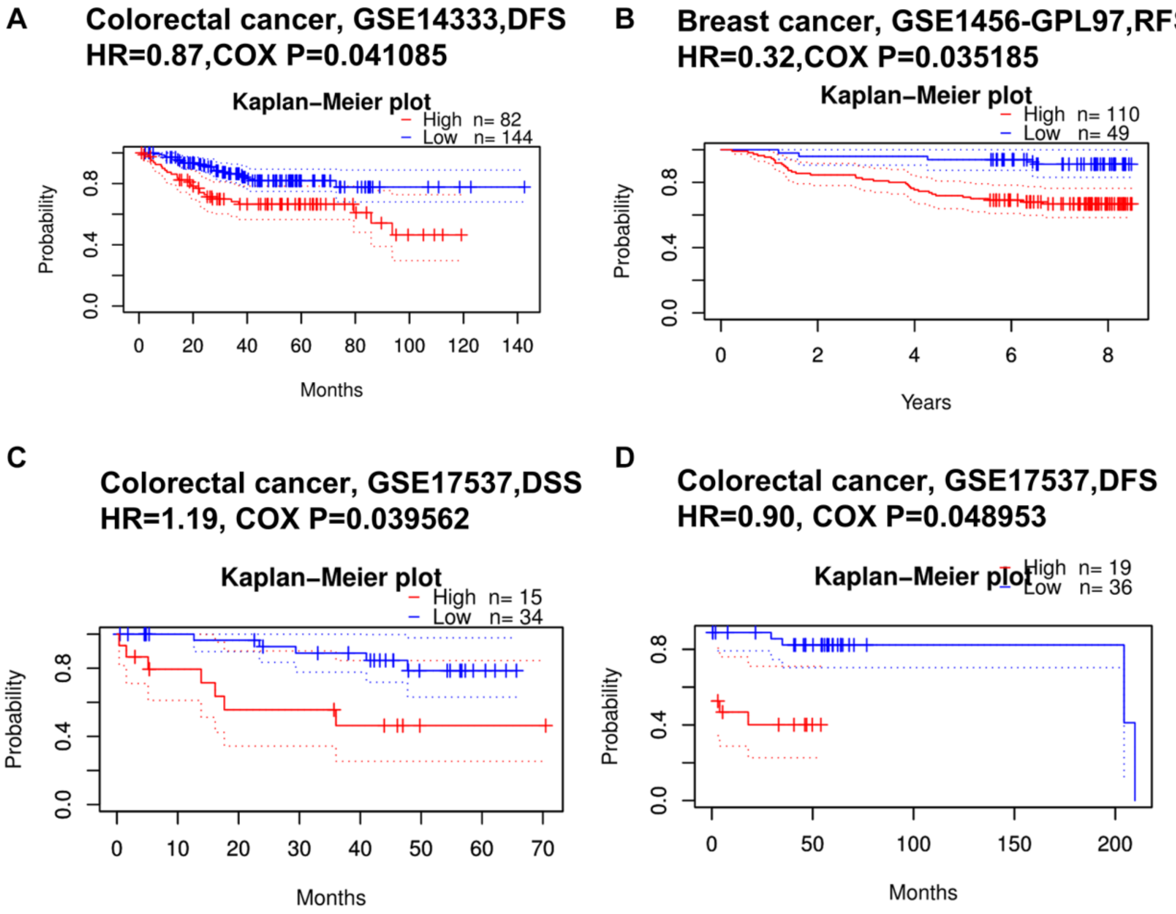

Supplementary Figure 5. The prognostic values of HOTAIR in different human cancers by PrognoScan database. (**A**) Disease free survival (DFS) curve of colorectal cancer; (**B**) Recurrence free survival (RFS) curve of breast cancer; (**C**) Disease-specific survival (DSS) curve of colorectal cancer; (**D**) Disease Free Survival (DFS) curve of colorectal cancer.

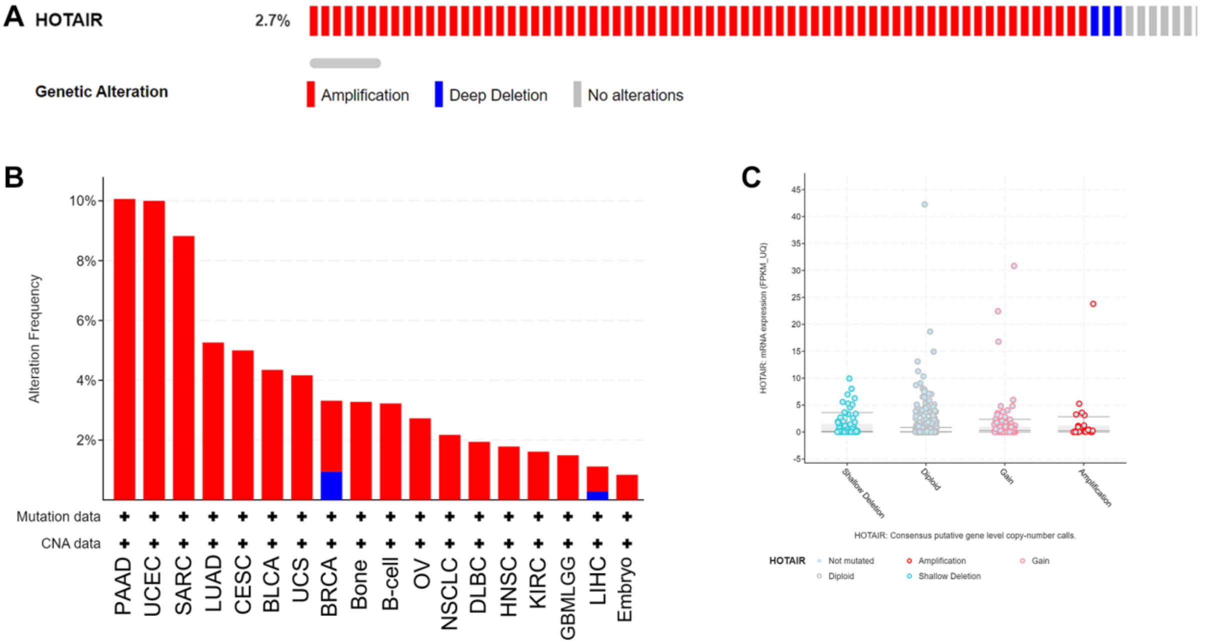

Supplementary Figure 6. The genetic alterations of HOTAIR. (**A**) Summary of HOTAIR structural variant, mutations, and copy-number alterations. (**B**) Alterations summary of HOTAIR in TCGA pan-cancer datasets. (**C**) The alteration types of HOTAIR in pan-cancer.

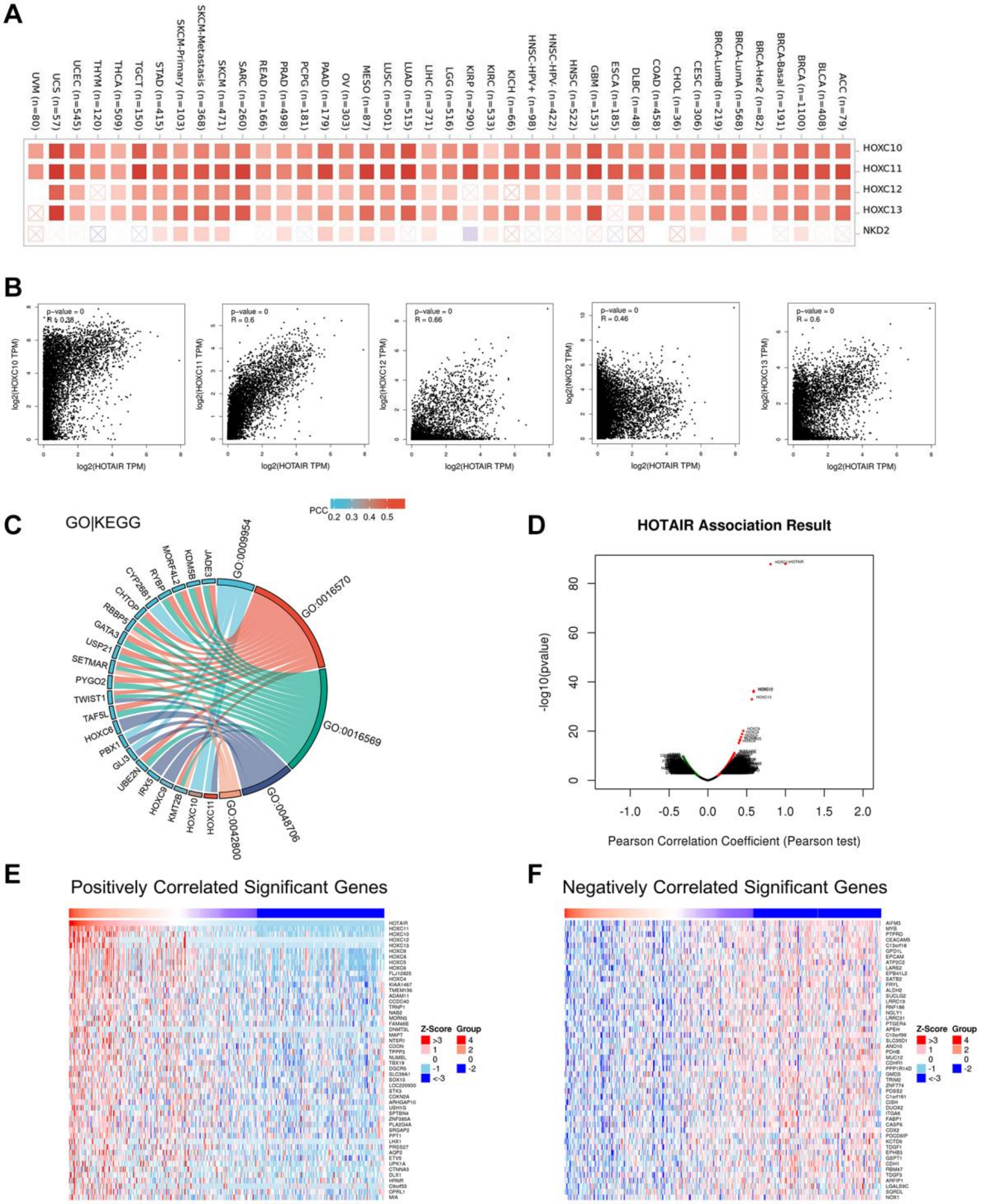

Supplementary Figure 7. Enrichment analysis HOTAIR-related gene. (**A**) Heatmap representation of the expression correlation between HOTAIR and HOXC10, HOXC11, HOXC12, HOXC13, and NKD2 in TCGA tumors. (**B**) The correlation between HOTAIR and selected genes from GEPIA2 tool. (**C**) GO|KEGG analysis based on HOTAIR-related genes. (**D**) Volcano plot analysis of highly correlated genes of HOTAIR tested by Pearson test in COAD cohort using LinkedOmics. (**E**, **F**) Top 50 positive co-expression genes (**E**) and negative co-expression genes (**F**) of HOTAIR in heat map in COAD.
