# Supplementary material for: Long non-coding RNA HOTAIR: from pan-cancer analysis to colorectal cancer-related uridine metabolism
Source: Aging (Albany NY). 2024 May 1;16(9):7752–73. doi: 10.18632/aging.205781 (PMC11132002; doi:10.18632/aging.205781)

**Supplementary Data 1**

**siRNA**

| Gene | Sequence（ 5' → 3' ） |
| --- | --- |
| si1-HOTAIR | AAAUCCAGAACCCUCUGACAUUUGC |
| si2-HOTAIR | UUAAGUCUAGGAAUCAGCACGAAGC |
| si-EZH2 | AAGAGGUUCAGACGAGCUGAUTT |

**Primer sequences**

| Gene | Sequence（ 5' → 3' ） |
| --- | --- |
| HOTAIR | F: CAGTGGGGAACTCTGACTCG |
|  | R: GTGCCTGGTGCTCTCTTACC |
| UPP1 | F: ATGGGCATTCCTTCTATC  R: GACAATCTGCTCAAACTC |
| UPP1-promoter | F: GGCTTGTCTGCGGGATG  R: CGGAGCACTCGAATGAGG |
| GAPDH | F: GAAGGTGAAGGTCGGAGTC |
|  | R: GAAGATGGTGATGGGATTTC |

**shRNA**

**OE-UPP1**


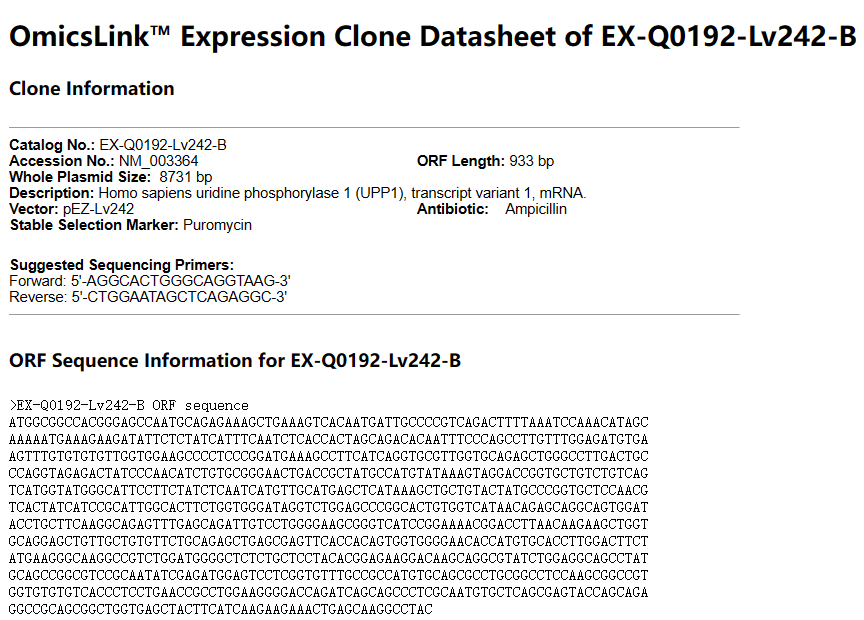


**sh1-UPP1**


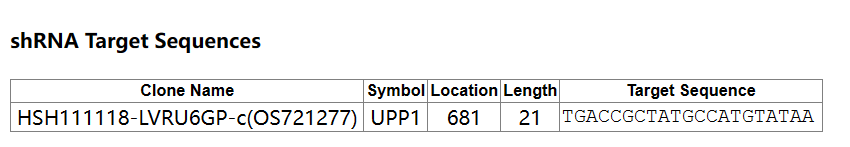


**sh2-UPP1**


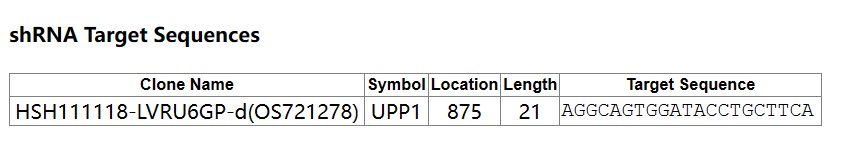


**sh3-UPP1**


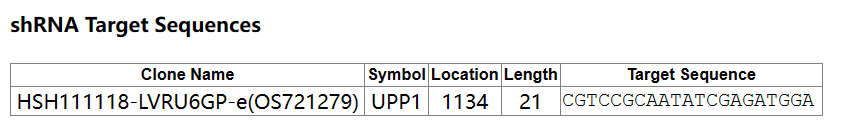

Supplement: Supplementary Data 1 [file aging-16-205781-s002.docx]
